# The Metallome of Lung Cancer and its Potential Use as Biomarker

**DOI:** 10.3390/ijms20030778

**Published:** 2019-02-12

**Authors:** Belén Callejón-Leblic, Ana Arias-Borrego, Antonio Pereira-Vega, José Luis Gómez-Ariza, Tamara García-Barrera

**Affiliations:** 1 Department of Chemistry, Faculty of Experimental Sciences, University of Huelva, Campus de El Carmen, Research Center on Health and Environment (RENSMA), 21007 Huelva, Spain; belen.callejon@dqcm.uhu.es (B.C.-L.); ana.arias@dqcm.uhu.es (A.A.-B.); 2 Pneumology Area of Juan Ramón Jiménez Hospital, 21005 Huelva, Spain; antonio.pereira@gmail.com

**Keywords:** lung cancer, metals, homeostasis, ICP-MS, elemental ratios, classification

## Abstract

Carcinogenesis is a very complex process in which metals have been found to be critically involved. In this sense, a disturbed redox status and metal dyshomeostasis take place during the onset and progression of cancer, and it is well-known that trace elements participate in the activation or inhibition of enzymatic reactions and metalloproteins, in which they usually participate as cofactors. Until now, the role of metals in cancer have been studied as an effect, establishing that cancer onset and progression affects the disturbance of the natural chemical form of the essential elements in the metabolism. However, it has also been studied as a cause, giving insights related to the high exposure of metals giving a place to the carcinogenic process. On the other hand, the chemical species of the metal or metallobiomolecule is very important, since it finally affects the biological activity or the toxicological potential of the element and their mobility across different biological compartments. Moreover, the importance of metal homeostasis and metals interactions in biology has also been demonstrated, and the ratios between some elements were found to be different in cancer patients; however, the interplay of elements is rarely reported. This review focuses on the critical role of metals in lung cancer, which is one of the most insidious forms of cancer, with special attention to the analytical approaches and pitfalls to extract metals and their species from tissues and biofluids, determining the ratios of metals, obtaining classification profiles, and finally defining the metallome of lung cancer.

## 1. Introduction

Cancer is a multifaceted disease and over time, metals, metabolites, and microbes, among others, have been proven to be critically involved in the onset and progression of this disease. In particular, lung cancer (LC) is the second most prominent type of cancer in the world, the first cancer death cause [1], and the five-year survival rate is only 15% [2]. 

In relation to metals, there are two different points of view to explain their role in the organism during the carcinogenic process, because they can be considered a cause or an effect. The first one is based on the hypothesis that cancer onset and progression disturb the natural chemical form of the essential element in the metabolism. The second one considers that elements are involved in the carcinogenic process because they are a consequence of their high exposure. Moreover, metals also contribute to cancer progression and metastasis [3,4,5,6,7]. On the other hand, the majority of works focus on the estimation of a deficiency state or excess and into a lesser extent in the unbalance episodes in which one element affects the function of others.

Approximately one-third of proteins need the presence of metals as cofactors to develop their function (metalloproteins), and in general, more than 50% of the proteins are influenced by metals [8]. Likewise, metals are responsible for the structure or catalytic properties of proteins, and their link to a molecule is usually determined by the genome [9]. Moreover, elements are essential to maintain human homeostasis and play key roles in systems biology, participating in many cellular processes. Essential metals, which are only required in trace amounts, are crucial for the function of numerous enzymes that are required for fundamental biochemical processes, and non-essential metals can alter the function of some enzymes as well [10]. However, metals are also involved in numerous biological processes such as the transport of oxygen in the blood; this process is mediated by hemoglobin, which contains Fe [11]. The deficiency or excess of any of these elements can lead to disease (e.g., anemia) or deleterious toxic effects, inflammation [12], and cancer [13]. For this reason, the levels of essential metals have to be carefully balanced, and a homeostatic state has to be maintained within the body [12]. 

On the other hand, a number of non-essential elements can also have important implications on human health. In this way, environmental exposure to As, Cd, Pb, and Ni has carcinogenic consequences [13] due to the activation of oncogenic signaling pathways [14,15] and oxidative stress [13,16,17]. The concentration and activity of enzymatic antioxidants are related to the concentration of elements [18]. Likewise, a disturbed redox status in lung cancer patients has been proven to be related to alterations of Zn, Mn, and Cu, since they regulate the levels and activities of antioxidants (mainly enzymatic ones), and the disturbed redox status may be critical to lung carcinogenesis. In this sense, these metals are cofactors or ions stabilizing the molecular structure of superoxide dismutase (SOD), which is an endogenous antioxidant [19]. Moreover, the type of biological fluid influences both alterations in the metal profile and relationships with redox status parameters [18]. 

Although the interaction of elements has not been extensively researched, it is known that metals can influence the function of others through antagonistic or synergistic mechanisms, as is the case of the competitive interaction of Zn and Cd for many enzymes due to their very similar atomic structure [20]. It has been pointed out that this interaction has dramatic effects on many zinc-containing enzymes that are involved in important biological processes leading to cancer onset [21]. Another important interaction between metals that has been described in the literature in connection with lung cancer is that of Cu and Zn. Decreased Zn and elevated Cu concentrations (high Cu:Zn ratio) in serum are the most successfully used predictor variables to distinguish between lung cancer (LC) and healthy controls (HC) [18,22,23,24]. Cu is an essential trace element in the diet, and has a pivotal role in enzymes related to oxidative metabolism, since it is needed for the interconversion of cupric and cuprous ions [25], but also contributes to the generation of free radicals [25]. Zn is a constituent of over 300 enzymes that play vital roles in gene expression [26]. Increased levels of Cu in serum of LC have been related to tumor progression through angiogenesis (the formation of new capillaries from existing blood vessels) to meet the nutritional requirements of a growing tumor [27,28]. Decreased levels of Zn have been related to alterations in the cell cycle and apoptosis [29,30], and the inability to block the further absorption of Cu [31]. Low serum Zn concentrations may affect protein synthesis and delay the repair processes of the body because of possible influences of depleting Zn-binding protein and metalloenzymes [32]. Moreover, it seems to be correlated with lymphocyte dysfunction [33]. Moreover, Zn^2+^ acts a cofactor for histone deacetylases (HDACs), which are a family of enzymes that are responsible for protein acetylation and deacetylation. An anomalous histone acetylation, and consequently an alteration in the expression of HDACs, is related to cancer [34,35,36,37,38,39] Likewise, an overexpression of HDAC1 and HDAC2 has been found in LC [40], and HDAC8 knockdown leads to the inhibition of cell proliferation in lung and other types of cancer [41]. Thus, an upregulation in concentrations of Zn^2+^ could be related to perturbation in the action of HDACs. 

Synergistic interactions have also been described as in the case of Zn and Se, whose depressed levels seem to be involved in carcinogenesis by increasing DNA damage and oxidative stress, decreasing antioxidant defense capacity, inhibiting DNA repair mechanisms, and declining immune function [42,43]. The chemopreventive character of Se for most cancers has been extensively reviewed, mostly in relation to its antioxidative role via glutathione peroxidase (GSH-Px) [44]. It seems that the depressed levels of Se in serum from LC patients might be a consequence of increased uptake by tumoral tissues in response to oxidative stress mediated by free radicals, since GSH-Px is required by the tumor tissues to prevent the damage caused by free radicals. Se is also involved in inhibiting angiogenesis [45] and enhancing the immune response [46], and is a well-documented antagonist against toxic metals [47]. Se also controls the unnecessary proliferation of cells [48] and inhibits the activation of certain transcription factors [49]. Mn superoxide dismutase (Mn-SOD), as a single superoxide radical scavenger in mitochondria, may have a key role in preventing cells as an antioxidant and tumor suppressor [50]. In the lungs, Mn-SOD is considered to be of critical importance for antioxidant defense [51]. A number of studies have defined associations between the Mn-SOD Ala16Val polymorphism and different cancer types [52,53,54]. It has been stated that there is a disruption in Mn homeostasis during cancer development, resulting in mobility from one compartment to other [18].

Ni is a carcinogenic agent that has been implicated as a pulmonary carcinogen in tobacco smoke [55]. Nickel causes oxidative DNA damage. [56]. Cr is associated with glucose and lipid metabolism, protein synthesis, and other important physiological functions [57]. On the other hand, the hexavalent form of chromium and its containing compounds are well-established lung carcinogens. Chronic exposure of the normal human epithelial cells is able to induce malignant cell transformation, which is the first stage of metal carcinogenesis [58]. Some authors have reported on the anticancer properties of V, and showed that the complexes of these metals are the new metal-based drugs that are used in the treatment of several cancers, such as lung cancer [59]. Other authors have demonstrated the influence of vanadium compounds in the cytotoxicity of some ligands in human lung cancer-cultured cells [60].

The “metallome” can be defined as the ensemble of all the biomolecules in a system, which bind a given metal ion or an inorganic element, or are affected by that element. A number of subsets of a metallome can then be defined based on the nature of the biomolecules interacting with the metal ions and the inorganic elements in general [61]. Williams defined the “metallome” as the distribution of elements, or concentration at equilibrium of free metallic ions or free elements in a cellular compartment, cell, or organism [62]; the metallome is related to the identity and/or quantity of metals/metalloids and their chemical species [8,63,64]. In this way, metallomics considers that the identification of a metal cofactor in a protein is critical to finally assigning its function as well as placing it in the context of known cellular pathways [65,66]. In metallomics, metal or metalloids are used as tags or heteroatomic markers to measure metallomolecules in complex matrices, usually by means of a chromatographic separation step coupled to inductively coupled plasma mass spectrometry (ICP-MS) [67,68,69]. On the other hand, considering that chemical species can be defined as “the specific forms of an element defined to isotopic composition, electronic, or oxidation state and/or complex or molecular structure” [70], metallomics, metal-metabolomics, and chemical speciation are completely imbricated.

## 2. Metal Dyshomeostasis in Lung Cancer: Biofluids and Tissues

The relationship between metal exposure and lung cancer (LC) has been deeply studied, especially in occupational medicine and environmental studies. The use of metals for diagnosis is more scarce, but several authors have classified lung cancer patients and healthy people using metals content in tumor tissues [71,72], serum, hair [24,73], urine [74], and bronchoalveolar lavage fluid (BALF) [75], because they found statistically significant differences from the normal distribution of elements for the diagnosis of LC. Then, in the literature, it is possible to find levels of essential and non-essential elements in different biofluids and tissues of lung cancer (LC) patients, for example in plasma [76,77], serum [18,24,42,75,78], urine [74,75,79], pleural effusion [80], or hair [24,73], and recently in bronchoalveolar lavage fluid (BALF) for the first time [75]. Table 1 has a collection of the typical concentrations in tissues and biofluids of LC patients and HC that can be found in the literature. 

It is well-known that the use of biomarkers such as metals in hair, nails, blood (serum, plasma, or whole blood) or urine can be used to detect abnormal levels in the human body as a consequence of acute (biofluids) or long-term exposure (hair and nails). The type of biosample used in the study is also important, because metal profiles and their relationships with redox status parameters are different [18]. Likewise, serum seems to be better for Cu and whole blood for Mn, while in the case of Zn, it is not relevant. For the determination of the relationships between trace elements and redox status parameters, authors have recommended the use of different samples [18]. On the other hand, there are several papers describing the analysis of metals in bronchoalveolar lavage fluid [83,84], but only one is related to lung cancer patients [75]. 

BALF is obtained during the exploratory study of patients with lung diseases, and provides constituents’ information on the cellular and biochemical epithelial surface of the lower respiratory tract through the instillation and later aspiration of liquid in one or more lung segments. It is estimated that BALF samples take a million cells (1% of the lung surface) to yield about one ml of pulmonary secretions in the actual total recovered liquid [85]. Since BALF is in close interaction with lung tissue, it is a more representative sample of lung status than other biofluids such as blood or urine.

In serum, decreased Zn and elevated Cu concentrations (high Cu:Zn ratio) are the most common biomarkers of LC [18,24,42,81,82,86,87]. The concentration of Cu [88] and the Cu:Zn ratio [22,77] have also been demonstrated to be increased in the plasma of LC patients. The concentration of copper is related to the cancer state and localization, and is primarily found in serum (95% as part of the oxidative enzyme ceruloplasmin, and the remainder is loosely bound to albumin) [89]. On the other hand, low serum levels of Zn are usually correlated with high levels of Zn in tumor autopsy, which suggest that the metabolic requirements of this element by LC cells are taken from serum [90]. Ren et al. also found that Cu, Cr, P, and Zn were found to be significantly different between LC and HC [24]. As can be seen in Table 1, there are some contradictory results regarding the decreased concentration of Zn, which in some papers in the literature have been found to be increased in serum, urine, and BALF. However, the most important fact is that the Cu:Zn ratio is increased. 

Lin et al. [76] found higher levels of Mn in serum from the LC group, but there are contradictory results [18,42]. Among other elements, increased concentrations of Ti, Ni, and Cu, but lowered concentrations of V, Cr, Mn, Fe, Co, Zn, Se, and Br have been determined in the serum of LC patients [42].

However, other studies revealed higher concentrations of V in the serum of LC patients [75]. Finally, Cr is another element that is usually increased in the serum of LC patients [24,75,76].

The determination of 11 elements in the BALF of LC patients demonstrated that increased levels of Mn can be used as a biomarker, since it showed a VIP (Variable Importance on the Projection) value of 1.29, a fold change of 1.5, a p-value of 0.003, and an AUC (Area Under the Curve) value of 0.75 [75]. Other authors also reported decreased levels of manganese in BALF from patients with diffuse lung diseases [91] or calves with mycoplasma bronchopneumonia [92]. On the other hand, the determinations of metals in the pleural effusion (PE) of smokers with lung cancer revealed lower Zn concentrations [80]. On the other hand, an adequate Zn intake has been associated with a decreased risk of smoking-associated obstructive lung disorders [93]. Moreover, low Zn levels may result in high glucose levels in the PE of smokers with LC due to a decreased glucose metabolism [80].

In urine, Tan et al. demonstrated that Fe, Mn, and Al were significantly decreased in the urine of LC patients, while Se, Ni, Cu, Zn, Cd, and Cr increased [74,79]. Voyatzoglou et al. [23] reported that patients with lung cancer and hyperzincuria had more widespread disease and a shorter life expectancy compared with patients with normal urinary zinc levels. On the other hand, a recent study revealed that the concentration of Cd, which is a recognized carcinogen, was increased in the urine of LC patients [75]. 

The concentration of elements has also been determined in the hair of LC patients versus HC, showing that the levels of Zn, Mg, Fe, and Se were higher in LC patients, while Ca, Sn, and Na were lower [73]. Other authors also described that Al, B, Cr, P, and Sr were significant variables for the classification between LC versus HC [24]. 

The study of metals in the tumor tissues of lung cancer patients showed decreased Fe and Mn levels in malignant versus normal lung tissue giving the best classification. These two elements correctly identified 10 of the 13 malignant tissues with an accuracy of 77%. The inclusion of other elements, especially Cu, but also others such as Ca, V, Fe, Cu, Zn, Se, Br, Sr, Hg, As, and Mo resulted in the correct grouping of 100% of samples [71]. Other authors found that the concentrations of P, Ti, and Pb increased in LC patients versus HC, while the levels of Ca, Fe, Cu, and Zn decreased [72]. Other authors reported that the levels of studied elements showed a trend of accumulation in cancer tissues [88]. Moreover, several authors found differences between concentrations of several metals according to sex [72] and age [94] in the tumor tissues of LC patients. 

In relation with the analytical techniques used in these studies, most elemental determinations in biofluids have been performed by atomic absorption spectroscopy (AAS) [81,82,83,87,91], particle-induced X-ray emission [95], energy-dispersive X-ray fluorescence [96], inductively coupled plasma atomic emission spectroscopy (ICP-AES) [24,74,79], or inductively coupled plasma mass spectrometry (ICP-MS) [73,76,77,80,84]. The main advantage of using an ICP-MS equipped with a triple quadrupole is the elimination of interferences by operating in either standard single quadrupole (SQ) mode or triple quadrupole (ICP-QQQ-MS). For instance, in the case of selenium, great advantages can be obtained with an ICP-QQQ-MS, because the signal of ^80^Se in oxygen mode (^96^SeO^+^) would overlap with signals such as ^96^Zr^+^, ^96^Mo^+^, or ^96^Ru^+^ in a conventional ICP-MS, but with the ICP-QQQ-MS, this drawback is overcome through eliminating these elements in the first quadrupole [75]. 

## 3. Inter-Element Ratios and Correlations as Biomarkers of Lung Cancer

The importance of metal homeostasis and metals interactions in biology has been extensively investigated [47]. Regarding lung cancer disease, the majorities of works have focused on the estimation of a deficiency state or excess, and have examined the unbalanced episodes in which the excess of one element affects the function of other to a lesser extent. Although the interplay of elements is rarely reported, it is possible to find two different ways to evaluate the interactions of metals based on the metals’ ratios and correlation coefficients. As commented before, the Cu:Zn ratio has been demonstrated to be increased in the serum [18,23,24,42,81,82,86,87], plasma [22,77], whole blood [18], urine [23], hair [24], and pleural effusion [80] of lung cancer patients against healthy controls. 

A recent study of our group demonstrated that the ratios between elements were important biomarkers for lung cancer disease, including: V/Mn, V/Pb, V/Zn, Cr/Pb in serum, Cr/Cd, Mn/Cd, V/Cd, Co/Cd, Cd/Pb in urine and V/Cu in BALF. These ratios reflect the dyshomeostasis of metals that takes place. Taken into account that cancerous and normal tissues differ significantly in their cell composition, other authors measured for each metal the ratio to K, since it is the most abundant intracellular cation [88]. The results showed that the Zn/K ratio was reduced in LC and HC, whereas Cu/K slightly increased [88]. The ratio between Cd and Zn has also been found to be different between smokers and nonsmokers, and it is also different among smokers for several different diseases and cancers [97].

On the other hand, several metals are correlated to others, suggesting also the existence of an interconnected homeostasis in lung cancer [75], as can be seen in Table 2. In this way, selenium is positively correlated with zinc in BALF samples of LC. The antioxidant properties of selenium are well-known, and alterations of this element can be related to oxidative stress. In this work, selenium is positively correlated with zinc in BALF samples, which is implicated in glucometabolic disorders [98]. Cobalt and copper are also positively correlated in BALF. The function of cobalt in the body is to be a cofactor of vitamin B12, but in the form of labile ions, it is able to generate reactive oxygen species, such as copper and iron [99]. 

## 4. Selenometabolites and Selenoproteins and Their Role in Lung Cancer

One of the most known elements for its beneficial effects on health and antagonistic protective action against many pollutants is selenium. However, selenium is essential only in a narrow range of concentrations, and the chemical form also determines its essentiality/toxicity. The active center of selenoproteins (with selenocysteinyl residues) such as glutathione peroxidase and other selenoenzymes is (–SeH), but selenium can also be present in the living body as selenium-containing proteins (with selenomethionyl residues, ie selenoalbumin), inorganic selenium, selenoamino acids, and methylated selenium [100]. The selenium species that is most abundant in the bloodstream is selenoprotein P (SeP), and its concentration is a good indicator of Se status in humans, while extracellular glutathione peroxidase (eGPx) is a complementary marker of selenium status in several clinical studies [101]. Moreover, Se-proteins are interrelated, because Se bound to albumin (SeAlb) is assumed to be transported to the liver for the new synthesis of SeP and GPx, which are then released into the bloodstream. The concentration of selenium in human serum is about 90 ng·g^-1^, and this elements occurs as SeP > Selenoalbumin (SeAlb) > GPx > SeO_3_^2−^ [102]. As previously commented, selenium is part of the enzyme glutathione peroxidase (GPx), which is considered the main agent against free radicals, as an anticancer, and inhibits the toxicity of some xenobiotics [103]. Until now, only 25 selenoproteins are known in humans, including four types of glutathione peroxidase (GPX1, GPX2, GPX3, and GPX4) and two thioredoxin reductases (TXNRD1, TXNRD2), but the function of many others is still unknown [104].

Selenium has been studied previously in relation to cancer from several points of view. On the one hand, it has been shown that there is a decrease in the risk of lung cancer in populations where selenium levels are high [105]. To this end, selenium levels in plasma samples from 372 lung cancer patients were determined by SeP determination using the ELISA method [105]. In another study, serum selenium levels were determined in 95 patients with lung cancer and the genotypes of four selenoprotein genes (GPX1, GPX4, TXNRD2, and SEP15) were determined, demonstrating that a selenium level less than 60 μg·L^−1^ is associated with a higher risk of lung cancer [106].

On the other hand, the chemopreventive character of selenium against cancer has been studied. In this way, it has been shown that selenium supplements reduce the mortality caused by several types of cancer, including lung cancer [107]. The molecular mechanism is not completely known, although different biological and biochemical processes have been identified [108], demonstrating that the effects observed at the molecular, cellular, and systemic levels include the expression of genes and the direct alteration of proteins [109]. The effects of selenium depend on its concentration, since low concentrations are absolutely necessary for cell growth, but moderate to high concentrations inhibit it. This inhibition of growth is tumor-specific, and selenium induces apoptosis in malignant cells at concentrations that do not affect the viability of normal cells [110]. Selenium species can affect carcinogenesis in different stages depending on the chemical species of selenium that is administered.

Several studies have suggested that polymorphisms in the selenoprotein gene of 15 kDa (SEP15) can alter the interaction of selenium with this protein, which is associated with an increase in susceptibility to various types of cancer [111], including LC [112]. The 15-kDa selenoprotein (SEP15) and other members of the thioredoxin family [113] are a type of catalytic agent that can regulate cellular redox reaction and reduce accumulated oxidative stress, which correlates with cell death and oncogenesis [114]. Finally, the relationship between the CASP8, MMP1, IL10, and SEPS1 genes and the risk of non-small cell lung cancer have also been demonstrated [115].

## 5. Chemical Species, Metal-Metabolites, and Metalloproteins

It is well-known that the chemical form of an element determines its toxicity and biological activity, as well as its mobility across different biological compartments. On the other hand, metallobiomolecules play key roles in the onset and progression of several diseases, in particular in lung cancer, and for this reason, they may be used as biomarkers of diagnosis and prognosis, as well as in therapies. Likewise, the design of the therapies based on metalloproteins is a growing research area [116]. For instance, the targeting of the zinc finger proteins by miRNAs, which act as a tumor suppressor by reducing cell proliferation, migration, and invasion as well as inducing apoptosis in human glioblastoma cell lines and glioma stem cells [117].

In this sense, there are few works related with the determination of metallobiomolecules in cancer, and even less in relation to lung cancer. In this context, the importance of metallothioneins (MT) in tumor formation, progression, and drug resistance has been proven [118]. For this reason, MTs may provide potential promising markers for cancer. MTs are high-content cysteine proteins, which play critical roles in homeostasis and protection against heavy metals, DNA damage, and oxidative stress, acting as antioxidants against free radicals and oxidative stress. Moreover, MTs control the homeostasis of the Cu/Zn ratio, which is essential in cell proliferation and differentiation [118]. Different isoforms of these metallothioneins (MTs) have been determined in the lung by studying related genes by means of the analysis of the reaction in the polymerase chain with reverse transcriptase (RT-PCR) [119,120]. The down-expression of MTs isoforms (MT1A, MT2A, MT1E, and MT1G), which are related to gene methylation, has been described in lung cancer [119].

On the other hand, the metalloprotein profiles in human plasma have also been described using a flow fractionation system with asymmetric flow field coupled to inductive coupling plasma with mass detector (AF4-ICP-MS). This study demonstrated that the profiles of Mn, Ni, Cu Zn, I, and Ba are different in patients with lung cancer and controls, and several metalloproteins have been identified by coupling nanochromatography and ion trap mass spectrometry with an electrospray ionization source/mass spectrometry [77]. In particular, the Zn metallobiomolecules have been studied in childhood brain tumors [117].

Under our knowledge, the speciation of elements should be performed in samples of LC patients. As discussed before, elements can be mainly present as labile ions or complexed with low molecular mass ligands, or in the form of metalloproteins. In a study of our group, an analytical metallomic approach based on the non-denaturing precipitation of proteins (NDPP) was optimized for the fractionation of high molecular mass (HMM) and low molecular mass (LMM) metal species, in order to distinguish between metal species that affect the biological activity and toxicological potential of the elements [75]. We concluded that several metals are good biomarkers when they are related to the labile ions, complexed with low molecular mass ligands, or in the form of metalloproteins (i.e., V and Cr in HMM and Cu in LMM), which were described for the first time. The analytical methodology was based on the use of an inductively coupled plasma triple quadrupole mass spectrometry (ICP-QQQ-MS), which has not been previously used in biofluids from LC patients [75].

## 6. Gaps and Future

Significant evidences can be found in the literature in relation to the key role of metals in the onset and progression of cancer, particularly in lung cancer. This open the possibilities of using them as biomarkers of early diagnosis, prognosis, or therapy. However, intense research should be performed in connection with the differentiation between chemical species, metalloproteins, and the interplay of elements on the basis of metals ratios and correlations. The Cu:Zn ratio has been demonstrated to be the best inter-element biomarker for lung cancer diagnosis in the majority of the samples, but recent studies have also demonstrated the importance of V/Mn, V/Pb, V/Zn, and Cr/Pb in serum; Cr/Cd, Mn/Cd, V/Cd, Co/Cd, and Cd/Pb in urine; and V/Cu in BALF, which reflect the dyshomeostasis of metals that takes place in LC. Important significant correlations can be found between elements in LC patients, such as for example between selenium and zinc, which is implicated in glucometabolic disorders, cobalt and copper, and copper and iron, among others. The influence of individual factors (i.e., sex, age, diet, etc.) in the concentration of metals that can be found in biofluids may be the main pitfall regarding the use of metals dyshomeostasis as biomarkers of lung cancer, and effort should be made in this direction to correctly establish a direct cause–effect link.

Powerful analytical techniques can be used for metal identification and quantification, mainly inductively coupled plasma mass spectrometry (ICP-MS) with single or preferably triple quadrupole mass spectrometer equipped with a collision/reaction cell for the elimination of interferences. The selection of the biofluid to be analyzed is critical, as well as the sample preparation procedure to isolate metal-containing fractions (high/low molecular mass fractions) or metallobiomolecules, for instance metalloproteins, of which 50% are influenced by metals. 

## Figures and Tables

**Table 1:** Typical concentrations of metals in biofluids and tissues of lung cancer (LC) patients.

Element	Sample	Average Concentration (µg·L^−1^)				FC (LC/HC)	*p*	Analytical Technique	Reference
		LC	SD	HC	SD				
Ag	Pleural effusion	0.2 *^,1^	-	0.18 *^,1^	-	1.11	N.S.^a^	ICP-MS	[80]
	Hair	0.547	0.696	0.722	1.416	0.76	*p* < 0.05 ^b^	ICP-MS	[73]
Al	Pleural effusion	91.1 *^,1^	-	199.3 *^,1^	-	0.46	N.S. ^a^	ICP-MS	[80]
	Hair	1879	2405	12820	4104	0.15	N.SP.	ICP-AES	[24]
	Hair	16.46	16.31	11.366	12.685	1.45	N.S. ^b^	ICP-MS	[73]
	Urine	189.11	58.94	220.8	147.3	0.86	*p* < 0.01 ^b^	ICP-AES	[79]
As	Pleural effusion	3.95 *^,1^	-	2.66 *^,1^	-	1.48	N.S. ^a^	ICP-MS	[80]
	Plasma	2.49	1.35	2.74	1.91	0.91	N.S. ^b^	ICP-MS	[76]
	Hair	0.458	1.269	0.558	0.742	0.82	N.S. ^b^	ICP-MS	[73]
Au	Hair	2.744	6.471	0.687	1.218	3.99	*p* < 0.05 ^b^	ICP-MS	[73]
B	Hair	277	386	1896	953	0.15	N.SP.	ICP-AES	[24]
	Plasma	65.79	38.06	70.13	35.81	0.94	N.S. ^b^	ICP-MS	[76]
	Hair	1.764	2.058	1.136	1.915	1.55	N.S. ^b^	ICP-MS	[73]
Ba	Hair	96,780	117,900	156,900	114,800	0.62	N.SP.	ICP-AES	[24]
	Plasma	7.78	5.98	6.42	5.07	1.21	N.S. ^b^	ICP-MS	[76]
	Hair	1.461	1.972	1.396	1.513	1.05	N.S. ^b^	ICP-MS	[73]
Be	Hair	0.012	0.017	0.038	0.074	0.32	*p* < 0.05 ^b^	ICP-MS	[73]
Bi	Hair	0.872	0.916	0.445	0.718	1.96	*p* < 0.05 ^b^	ICP-MS	[73]
Ca	Serum	75,620	11,140	93,780	6851	0.81	N.SP.	ICP-AES	[24]
	Hair	969,300	944,700	1,215,000	822,200	0.80	N.SP.	ICP-AES	[24]
	Hair	68.25	61.33	30.812	18.809	2.22	*p* < 0.05 ^b^	ICP-MS	[73]
Cd	Hair	51	48	245	501	0.21	N.SP.	ICP-AES	[24]
	Hair	0.209	0.176	0.316	0.426	0.66	*p* < 0.05 ^b^	ICP-MS	[73]
	Urine	10.06	2.66	6.69	5.11	1.50	*p* < 0.01 ^b^	ICP-AES	[79]
	Serum	0.18 ^1^	0.04	0.07 ^1^	0.00	2.50	N.S. ^c^	ICP-QQQ-MS	[75]
	Urine	1.58 ^1^	0.21	0.55 ^1^	0.07	2.86	*p* < 0.05 ^c^	ICP-QQQ-MS	[75]
	BALF	0.14 ^1^	0.04	0.08 ^1^	0.02	1.82	N.S. ^c^	ICP-QQQ-MS	[75]
Ce	Hair	2.724	8.11	0.958	2.886	2.84	*p* < 0.05 ^b^	ICP-MS	[73]
Co	Pleural effusion	0.24 *^,1^	-	0.17 *^,1^	-	1.41	N.S. ^a^	ICP-MS	[80]
	Hair	33	36	57	47	0.58	N.SP.	ICP-AES	[24]
	Hair	3.131	11.057	0.392	0.467	7.99	*p* < 0.05 ^b^	ICP-MS	[73]
	Serum	0.46	0.07	0.27	0.07	1.71	*p* < 0.05 ^c^	ICP-QQQ-MS	[75]
	Urine	0.43	0.02	0.65	0.05	0.67	*p* < 0.05 ^c^	ICP-QQQ-MS	[75]
	BALF	0.008	0.003	0.001	0.001	5.60	*p* < 0.05 ^c^	ICP-QQQ-MS	[75]
Cr	Pleural effusion	1.37 *^,1^	-	0.98 *^,1^	-	1.40	N.S. ^a^	ICP-MS	[80]
	Serum	1706	1326	111	164	15.37	N.SP.	ICP-AES	[24]
	Hair	ND	-	814	401	-	N.SP.	ICP-AES	[24]
	Plasma	33.4	7.64	31.47	8.55	1.06	N.S. ^b^	ICP-MS	[76]
	Hair	2.492	4.021	0.934	1.016	2.67	*p* < 0.05 ^b^	ICP-MS	[73]
	Urine	44.61	14.44	23.08	20.52	1.93	*p* < 0.01 ^b^	ICP-AES	[79]
	Serum	0.96	0.15	0.51	0.21	1.89	*p* < 0.05 ^c^	ICP-QQQ-MS	[75]
	Urine	1.90	0.17	1.91	0.27	0.99	N.S. ^c^	ICP-QQQ-MS	[75]
	BALF	0.30	0.07	0.19	0.04	1.58	N.S. ^c^	ICP-QQQ-MS	[75]
Cs	Hair	0.264	0.284	0.246	384	1.07	N.S. ^b^	ICP-MS	[73]
Cu	Pleural effusion	850.2 *^,1^	-	910.5 *^,1^	-	0.93	N.S. ^a^	ICP-MS	[80]
	Serum	1392	278	929	232	1.50	N.SP.	ICP-AES	[24]
	Hair	9827	1526	11,540	1237	0.85	N.SP.	ICP-AES	[24]
	Plasma	1256.3	214.7	1007	197.4	1.25	*p* < 0.01 ^b^	ICP-MS	[76]
	Serum	1518.8	69.9	1264.7	57.2	1.20	N.S. **	AAS	[81]
	Hair	24.45	18.47	15.753	16.73	1.55	*p* < 0.05 ^b^	ICP-MS	[73]
	Urine	73.25	24.04	29.93	21.87	2.45	*p* < 0.01 ^b^	ICP-AES	[79]
	Serum	1455.3	394.01	953.25	95.325	1.53	*p*≤0.05 ^b^	AAS	[82]
	Serum	1428.7	51.5	1251.1	56.0	1.14	*p* < 0.05 ^c^	ICP-QQQ-MS	[75]
	Urine	20.5	2.0	15.2	1.8	1.35	N.S. ^c^	ICP-QQQ-MS	[75]
	BALF	4.93	0.96	3.55	1.54	1.39	N.S. ^c^	ICP-QQQ-MS	[75]
Fe	Pleural effusion	747.7 *^,1^	-	1049 *^,1^	-	0.71	N.S. ^a^	ICP-MS	[80]
	Serum	2168	938	1988	913	1.09	N.SP.	ICP-AES	[24]
	Hair	23,100	13,550	16,180	4619	1.43	N.SP.	ICP-AES	[24]
	Plasma	1298.2	642.6	1469.1	514.2	0.88	N.S. ^b^	ICP-MS	[76]
	Hair	9.65	6.64	25.052	22.93	0.39	*p* < 0.05 ^b^	ICP-MS	[73]
	Urine	309.32	85.05	310.4	242.1	1.00	N.S. ^b^	ICP-AES	[79]
	Serum	6151.7 ^1^	981.3	2947.9 ^1^	470.8	2.09	*p* < 0.05 ^c^	ICP-QQQ-MS	[75]
	Urine	50.5 ^1^	4.2	35.5 ^1^	3.6	1.42	*p* < 0.05 ^c^	ICP-QQQ-MS	[75]
	BALF	21.7 ^1^	5.1	39.8 ^1^	20.1	0.55	N.S. ^c^	ICP-QQQ-MS	[75]
Ga	Hair	0.288	0.59	0.25	0.23	1.15	*p* < 0.05 ^b^	ICP-MS	[73]
Hg	Hair	1.233	1.367	0.585	0.713	2.11	*p* < 0.05 ^b^	ICP-MS	[73]
K	Hair	15.32	18.66	10.701	9.25	1.43	*p* < 0.05 ^b^	ICP-MS	[73]
La	Hair	369	381	728	372	0.51	N.SP.	ICP-AES	[24]
Li	Hair	0.595	0.67	571	0.586	0.00	N.S. ^b^	ICP-MS	[73]
Mg	Serum	20,160	3770	24,580	2655	0.82	N.SP.	ICP-AES	[24]
	Hair	77,330	93,220	141,800	107,900	0.55	N.SP.	ICP-AES	[24]
	Hair	28.92	24.203	31.921	21.315	0.91	N.S. ^b^	ICP-MS	[73]
Mn	Pleural effusion	0.83 *^,1^	-	0.87 *^,1^	-	0.95	N.S. ^a^	ICP-MS	[80]
	Hair	2523	1898	1130	1714	2.23	N.SP.	ICP-AES	[24]
	Plasma	5.29	5.06	4.22	2.88	1.25	N.S. ^b^	ICP-MS	[76]
	Hair	1.82	2.16	1.144	1.119	1.59	N.S. ^b^	ICP-MS	[73]
	Urine	4.74	3.23	6.95	5.43	0.68	*p* < 0.01 ^b^	ICP-AES	[79]
	Serum	1.74 ^1^	0.08	1.14 ^1^	0.14	1.52	*p* < 0.05 ^c^	ICP-QQQ-MS	[75]
	Urine	3.48 ^1^	0.23	4.09 ^1^	0.58	0.85	N.S. ^c^	ICP-QQQ-MS	[75]
	BALF	0.69 ^1^	0.09	0.46 ^1^	0.06	1.51	*p* < 0.05 ^c^	ICP-QQQ-MS	[75]
Mo	Pleural effusion	1.23 *^,1^	-	1.05 *^,1^	-	1.17	N.S. ^a^	ICP-MS	[80]
	Hair	158	221	132	75	1.20	N.SP.	ICP-AES	[24]
	Serum	0.73 ^1^	0.22	0.43 ^1^	0.06	1.67	*p* < 0.05 ^c^	ICP-QQQ-MS	[75]
	Urine	32.8 ^1^	2.9	25.8 ^1^	3.5	1.27	N.S. ^c^	ICP-QQQ-MS	[75]
	BALF	0.11 ^1^	0.02	0.09 ^1^	0.02	1.14	N.S. ^c^	ICP-QQQ-MS	[75]
Na	Hair	23.316	30.458	21.378	15.79	1.09	N.S. ^b^	ICP-MS	[73]
Ni	Hair	117	168	454	907	0.26	N.SP.	ICP-AES	[24]
	Plasma	14.35	11.09	13.36	9.43	1.07	N.S. ^b^	ICP-MS	[76]
	Hair	1.126	0.8	0.58	0.547	1.94	*p* < 0.05 ^b^	ICP-MS	[73]
	Urine	59.378	8.21	21	12.7	2.83	*p* < 0.01 ^b^	ICP-AES	[79]
P	Hair	176,800	3,891,000	203,100	44,160	0.87	N.SP.	ICP-AES	[24]
	Serum	103,700	17,600	116,100	32,760	0.89	N.SP.	ICP-AES	[24]
Pb	Pleural effusion	0.63 *^,1^	-	1.04 *^,1^	-	0.61	N.S. ^a^	ICP-MS	[80]
	Hair	2364	2302	4476	7295	0.53	N.SP.	ICP-AES	[24]
	Plasma	10.61	5.63	9.31	5.94	1.14	N.S. ^b^	ICP-MS	[76]
	Hair	8.577	19.88	5.09	6.198	1.69	N.S. ^b^	ICP-MS	[73]
	Serum	1.54 ^1^	0.18	1.11 ^1^	0.11	1.38	N.S. ^c^	ICP-QQQ-MS	[75]
	Urine	7.37 ^1^	0.46	6.20 ^1^	0.48	1.19	N.S. ^c^	ICP-QQQ-MS	[75]
	BALF	0.47 ^1^	0.13	0.16 ^1^	0.02	2.98	*p* < 0.05 ^c^	ICP-QQQ-MS	[75]
Rb	Hair	0.47	0.753	0.251	0.3	1.87	*p* < 0.05 ^b^	ICP-MS	[73]
Rh	Hair	0.555	0.614	0.414	0.486	1.34	*p* < 0.05 ^b^	ICP-MS	[73]
Sb	Plasma	4.8	3.36	5.93	3.55	0.81	*p* < 0.05 ^b^	ICP-MS	[76]
	Hair	0.988	0.77	0.235	0.344	4.20	*p* < 0.05 ^b^	ICP-MS	[73]
Sc	Hair	0.134	0.545	0.027	0	4.96	*p* < 0.05 ^b^	ICP-MS	[73]
Se	Plasma	69.88	15.88	77.95	17.38	0.90	*p* < 0.05 ^b^	ICP-MS	[76]
	Hair	13.7	19.784	20.135	21.042	0.68	N.S. ^b^	ICP-MS	[73]
	Urine	15.026	8.33	5.36	2.78	2.80	*p* < 0.01 ^b^	ICP-AES	[79]
	Serum	191.9 ^1^	6.6	187.8 ^1^	5.9	1.02	N.S. ^c^	ICP-QQQ-MS	[75]
	Urine	51.6 ^1^	6.4	36.5 ^1^	4.5	1.42	N.S. ^c^	ICP-QQQ-MS	[75]
	BALF	3.54 ^1^	0.58	3.43 ^1^	0.52	1.03	N.S. ^c^	ICP-QQQ-MS	[75]
Sn	Pleural effusion	0.36 *^,1^	-	0.33 *^,1^	-	1.09	N.S. ^a^	ICP-MS	[80]
	Hair	33.94	24.72	21.509	19.48	1.58	N.S. ^b^	ICP-MS	[73]
Sr	Serum	753	209	762	253	0.99	N.SP.	ICP-AES	[24]
	Hair	3037	2663	4894	3889	0.62	N.SP.	ICP-AES	[24]
	Plasma	24.4	12.8	23.9	13.2	1.02	N.S. ^b^	ICP-MS	[76]
	Hair	1.91	1.914	1.455	1.68	1.31	N.S. ^b^	ICP-MS	[73]
Ti	Plasma	48.5	24.5	44.5	30.1	1.09	N.S. ^b^	ICP-MS	[76]
	Hair	12.64	19.128	2.079	4.46	6.08	*p* < 0.05 ^b^	ICP-MS	[73]
V	Pleural effusion	0.22 *^,1^	-	0.36 *^,1^	-	0.61	N.S. ^a^	ICP-MS	[80]
	Plasma	3.54	1.23	3.57	1.18	0.99	N.S. ^b^	ICP-MS	[76]
	Hair	1.83	2.29	1.047	1.74	1.75	*p* < 0.05 ^b^	ICP-MS	[73]
	Serum	0.17	0.04	0.05	0.02	3.77	N.S. ^c^	ICP-QQQ-MS	[75]
	Urine	1.34	0.09	1.31	0.09	1.02	N.S. ^c^	ICP-QQQ-MS	[75]
	BALF	1.38	0.03	1.24	0.04	1.11	*p* < 0.05 ^c^	ICP-QQQ-MS	[75]
Y	Hair	10	33	ND	-	-	N.SP.	ICP-AES	[24]
Zn	Pleural effusion	351.7 *^,1^	-	545.7 *^,1^	-	0.64	*p* < 0.05 ^a^	ICP-MS	[80]
	Hair	151,380	37,160	171,100	41,160	0.88	N.SP.	ICP-AES	[24]
	Plasma	702.4	129.2	772.2	191.4	0.91	*p* < 0.01 ^b^	ICP-MS	[76]
	Serum	836.87	45.77	934.93	84.99	0.90	N.S. **	AAS	[81]
	Hair	53.22	60.3	109.763	95.33	0.48	*p* < 0.05 ^b^	ICP-MS	[73]
	Urine	1519.8	194.8	568.9	544.7	2.67	*p* < 0.01 ^b^	ICP-AES	[79]
	Serum	1136.4	67.0	917.5 ^1^	75.5	1.24	*p* < 0.05 ^c^	ICP-QQQ-MS	[75]
	Urine	637.0	89.7	572.3 ^1^	115.1	1.11	N.S. ^c^	ICP-QQQ-MS	[75]
	BALF	35.1	13.4	15.0 ^1^	5.7	2.33	*p* < 0.05. ^c^	ICP-QQQ-MS	[75]
	Serum	29	12	33	14	0.88	N.SP.	ICP-AES	[24]
	Serum	784.56	130.36	902.24	130.76	0.87	*p* ≤ 0.05 ^b^	AAS	[82]

BALF: Bronchoalveolar Lavage Fluid; LC: Lung Cancer; HC: Healthy Control; ND: Not Detected; SD: Standard Deviation; FC: Fold Change. N.S.: Non significant, N.SP.: Not specified. Average Concentration: Arithmetic Mean, Gaussian Distribution, * Data expressed on the median.; ^1^: Non-parametric method; ^a^: Kruskal–Wallis test; ^b^: Student T-test; ^c^: Mann–Whitey U-test; ** Statistical test not specified.

**Table 2:** Significant correlations between elements in the biofluids and tissues of lung cancer patients.

	P	S	Ti	Cr	Se	Mn	Fe	Hg	V	Zn	Pb	Cu	Co	Mo
P		Tissue [72]												
S														
Ti				Tissue [72]	Tissue [72]									
Cr					BALF [75]	Tissue [72] Hair [73] Urine [75]			Serum [75]	BALF [75]				
Se								Tissue [72]		BALF [75]				
Mn							Tissue [72] Urine [75]			Serum [75]		BALF [75]		
Fe											Urine [75]			
Hg														
V											BALF [75]			
Zn														
Pb														
Cu													BALF [75]	
Co														BALF [75]
Mo														

BALF: Bronchoalveolar Lavage Fluid.
